# Acquired trichostasis in postoperative site: a case report

**DOI:** 10.1186/1757-1626-2-9310

**Published:** 2009-12-11

**Authors:** Deba P Sarma, Benjamin A Maertins, Eric E Santos

**Affiliations:** 1 Department of Pathology, Creighton University Medical Center, Omaha, NE, 68131, USA

## Abstract

**Introduction:**

Usual causes of a papule or nodule in a post-operative site after resection of a skin tumor are residual or recurrent tumor, proliferative scar, or suture granuloma with inflammation and granulation tissue. Inverted or trapped hair, an acquired trichostasis, has not been implicated as a cause in such cases, this is probably the first case reported in literature.

**Case presentation:**

A 31-year-old woman underwent an excision of a ruptured epidermal cyst of the left axilla. One month later, the previous excision site was re-excised secondary to a non-healing, inflamed papule in order to exclude recurrent epidermal cyst formation. Microscopic examination revealed that the cause of the papular lesion was acquired trichostasis, rather than a recurrent epidermal cyst.

**Conclusion:**

A papular or nodular lesion at a postoperative site may rarely be caused by acquired trichostasis and should be considered as one of the differential diagnosis.

## Case presentation

**Figure 1:**
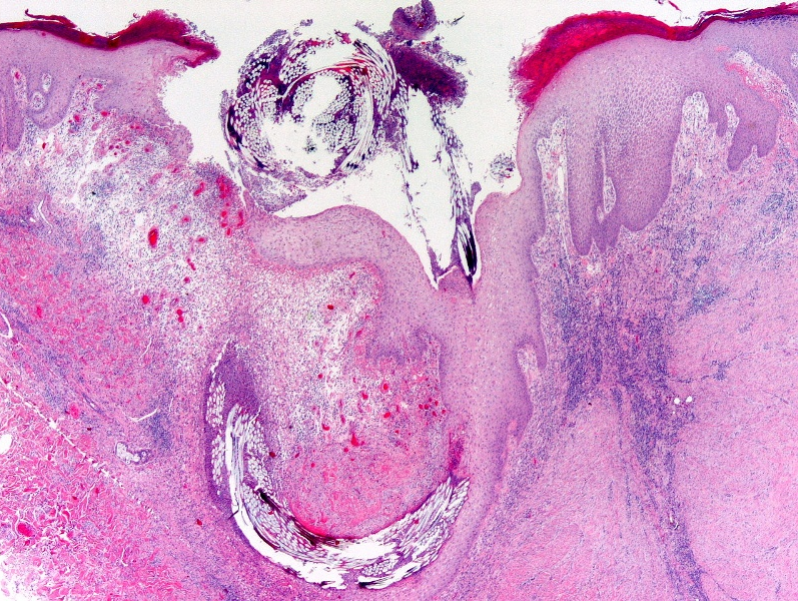
**Central cup-shaped invagination of a hair pore containing entangled hair**.

**Figure 2:**
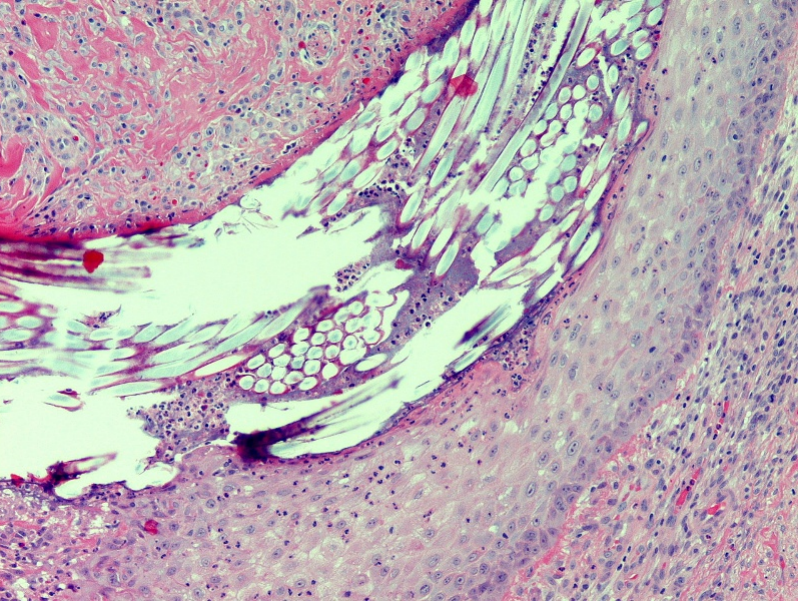
**Secondary trichostasis viewed at high power field demonstrates the cross section of a hair which may be confused with the appearance of a suture**.

**Figure 3:**
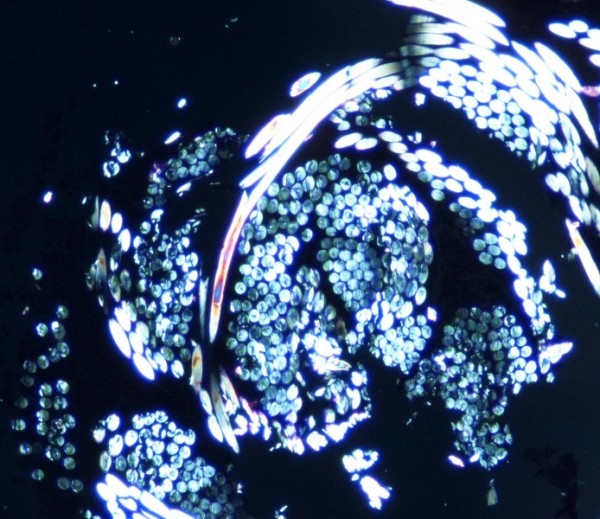
**Under polarized light, the entangled hair shows brilliant birefringence**.

A 31-year-old white American woman of European descent underwent an excision of a ruptured epidermal cyst of the left axilla. One month later, the previous excision site was re-excised secondary to a non-healing, inflamed papule in order to exclude recurrent epidermal cyst formation. On microscopic examination (Figure 1 and Figure 2), there was a central cup-shaped invagination of the epidermis containing a ball of refractile tissue and acute inflammatory exudates. Architecturally, the picture was that of a hair pore containing an entangled hair. The hair infundibulum extended downwards into the dermis containing the refractile hair shafts cut in cross sections. The dermis also showed granulation tissue, chronic inflammation, and scar formation. Under polarized light, the entangled hair revealed brilliant blue-red birefringence (Figure 3). There was no suture granuloma or residual epidermal cyst in the dermis.

## Discussion

Usual causes of a papule or nodule in a post-operative site after resection of a skin tumor are residual or recurrent tumor, proliferative scar, and/or suture granuloma with inflammation and granulation tissue. Inverted or trapped hair, an acquired trichostasis, has not been implicated as a cause in such cases.

The process of acquired trichostasis appears to begin at the post-operative site where the epidermal layer has been partially inverted as a result of suturing. An inversion of the epidermis blocks the hair pore from advancing to the surface of the skin and the hair gets trapped within the pore leading to entangled hair. The hair follicle may rupture in the dermis causing inflammation and fibrosis. Suture granulomas with fibrosis and scar are more common findings in postoperative sites. Trichostasis and suture granulomas may be found in the same location. Suture material is found within the dermal or subcutaneous fibroconnective tissue usually surrounded by foreign-body type giant cells. This is in sharp contrast to trichostasis where the entangled hair material is located within an epithelium-lined hair follicle. Moreover, the suture material on histological examination appears as a bluish, slightly refractile elongated structures with fragmentation.

By far, the most prevalent literature on trichostasis is that of trichostasis spinulosa which refers to retention of the vellus hair that protrudes from a single dilated ostium [1]. Clinically, the lesions of trichostasis spinulosa are multiple and can resemble comedones. There are two variants, pruritic versus non-pruritic types. Whereas the pruritic variant occurs on the limbs of young adults, the non-pruritic type, also referred to as the classical type, usually occurs on the face of middle aged individuals [2]. Although the pathogenesis for trichostasis spinulosa remains unknown, some studies speculate that pityrosporum and bacteria may induce follicular hyperkeratosis with vellus hair retention [3]. Postoperative trichostasis is entirely unrelated to trichostasis spinulosa.

## Consent

Written consent was obtained from the patient for publication of this case report and accompanying images. A copy of the written consent is available for review by the Editor-in-Chief of this journal.

## Competing interests

The authors declare that they have no competing interests.

## Authors' contributions

DPS conceived, and submitted the manuscript. BAM drafted the manuscript. EES revised the manuscript. All authors have read and approved the final manuscript.

## References

[B1] McKee PH Calonje E Grantner SR Pathology of the skin with clinical correlations 2005 Edinburgh: Elsevier Mosby 110

[B2] Young MC Jorizzo JL Sanchez RL Hebert AA Thomas DR King CA Trichostasis spinulosa Int J Dermatol 1985 24 9 575 580 10.1111/j.1365-4362.1985.tb05854.x 4066099

[B3] Chung TA Lee JB Jang HS Kwon KS Oh CK A clinical, microbiological, and histopathologic study of trichostasis spinulosa J Dermatol 1998 25 11 697 702 10.1111/j.1346-8138.1998.tb02486.x 9863280

